# Complete genome sequence of the avian paramyxovirus serotype 9 strain duck/Miyazaki/128/2021

**DOI:** 10.1128/mra.00060-24

**Published:** 2024-10-02

**Authors:** Hirohisa Mekata, Mari Yamamoto, Yuto Matsui, Ahmad Massoud Niazi, Kentaro Yamada, Tamaki Okabayashi, Se-Yeoun Cha, Hyung-Kwan Jang

**Affiliations:** 1 Center for Animal Disease Control, University of Miyazaki, Miyazaki, Japan; 2 Department of Avian Diseases, College of Veterinary Medicine, Center for Avian Disease, Jeonbuk National University, Iksan, South Korea; DOE Joint Genome Institute, Berkeley, California, USA

**Keywords:** paramyxovirus, avian viruses, APMV-9

## Abstract

Here, we report the complete genome sequence of the avian paramyxovirus serotype 9 strain duck/Miyazaki/128/2021, which was determined using the Illumina MiSeq platform. The position of the hemagglutinin-neuraminidase stop codon differed from that of the only other available completely sequenced prototype strain, duck/New York/22/1977.

## ANNOUNCEMENT

Avian paramyxovirus (APMV) serotype 9 is a member of the species *Orthoavulavirus newyorkense* and is classified in the family *Paramyxoviridae*. APMV-9 was first isolated in the 1970s (1); however, its analysis has been limited because it causes no clear symptoms in avian species (2). The first isolate, the duck/New York/22/1978 strain (accession no. NC025390), is the prototype of APMV-9 and the only strain for which a complete sequence has been registered (3). In this study, we report the second complete sequence of APMV-9.

The APMV-9 strain duck/Miyazaki/128/2021 was isolated from duck feces sampled in the Futatsudate, Miyazaki, Japan, in November 2021. The virus was isolated from 10-day-old embryonated chicken eggs (4), and the viral RNA was extracted using a MagLEAD system (Precision System Science, Chiba, Japan). Viral cDNA was synthesized using the PrimeScript II 1st strand cDNA Synthesis Kit (TaKaRa Bio, Kusatsu, Japan) with random hexanucleotide primers (5). After double-strand DNA synthesis and amplification, library preparation was performed using the QIAseq FX DNA Library Kit (Qiagen, Venlo, the Netherlands). Next-generation sequencing (NGS) was performed on a MiSeq instrument (Illumina, San Diego, CA, USA) using paired-end sequencing on a MiSeq Reagent Kit v3 (150 cycles). The adaptor sequences were trimmed, and low-quality reads were removed using CLC Genomic Workbench version 11 (Qiagen, Venlo, the Netherlands) with default parameters. A total of 4,305,930 reads were obtained, and they were *de novo* assembled using the software with default parameters. After confirming by BLASTn that the longest contig matched APMV-9, the reads were remapped to the APMV-9 strain duck/New York/22/1978. A single 15,414-bp consensus sequence was obtained without the 3′ or 5′ ends (total read count: 3,046,782; average coverage: 16,336). Both ends of the viral sequence were determined by the rapid amplification of cDNA end method according to previously reported methods (Table 1) (6). The sequences were determined using the BigDye Terminator version 3.1 Cycle Sequencing Kit, SeqStudio Genetic Analyzer (Thermo Fisher Scientiﬁc, Waltham, MA, USA) and ATGC-MAC version 7 software (Genetyx, Tokyo, Japan) and subsequently assembled into the sequence obtained by NGS.

**TABLE 1:** Primers to determine the sequences of the 5′ and 3′ ends of avian paramyxovirus serotype 9

Primer	Primer sequence*^a^*	Position from the end
VSP5-1	5′-TCATGGATTTTGCAACGGTCAC-3′	641–662
VSP5-2	5′-TGACACCGCTCCTATTGTTGAA-3′	461–482
VSP5-3	5′-TCATCACCTGTGAATGAGCACA-3′	353–374
VSP3-1	5′-GGGATCAACAAGGCACTCGA-3′	592–611
VSP3-2	5′-ACGTAGCAATTGCCCCAAGT-3′	272–291

^a^ All primers were designed in this study.

**Fig 1:**
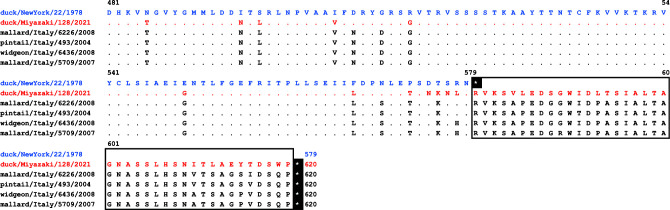
Alignment of the amino acid sequence of HN of avian paramyxovirus serotype 9. The blue letters indicate the prototype strain, duck/New York/22/1978, and the red letters indicate the strain presented in this study. White asterisks indicate stop codons.

The nucleotide sequence of the duck/Miyazaki/128/2021 consisted of 15,432 nucleotides, which is six nucleotides shorter than that of the prototype strain. The sequence similarity to the prototype strain was 91.3%, and the GC content was 45.0% by MEGA7 software (7). The amino acid sequence similarity of each protein was 96.5% (nucleoprotein), 87.8% (phosphoprotein), 96.7% (matrix protein), 96.7% (fusion protein), 94.1% (hemagglutinin-neuraminidase: HN), and 96.7% (large polymerase protein). The duck/Miyazaki/128/2021 strain had a mutation in the position of the HN stop codon of the prototype strain, resulting in 620 aa instead of 579 aa (Fig. 1). Four isolates in Italy from 2004 to 2008 (accession no. GU068584-GU068587) were partially sequenced on a 4,484-bp region, covering the entire length of the HN gene, and these HNs also consisted of 620 aa (8). Therefore, the stop codon of HN may have mutated in some of the APMV-9 field strains or as a result of repeated passages of the prototype strain. This study contributes to the understanding of APMV.

## Data Availability

The complete genomic sequence of the APMV-9 strain duck/Miyazaki/128/2021 has been deposited in GenBank under accession number LC790477. Sequence data files have been deposited in the DDBJ Sequence Read Archive under accession number DRR519924.
